# Elderly unstable distal radius fractures a prospective cohort study of bone substitutes-augmented percutaneous pinning

**DOI:** 10.1186/s12891-022-05202-2

**Published:** 2022-03-12

**Authors:** Amr Abdel-Mordy Kandeel

**Affiliations:** Department of Orthopedics & Traumatology, Faculty of Medicine, Menoufia University, Gamal Abdel-Nasser Street, Shebien El-Kom, Menoufia Governorate Egypt

**Keywords:** Bone substitutes-augmented distal radius fixation, Elderly distal radius fractures, K-wire fixation of distal radius fractures, Metaphyseal void of distal radius fractures, Percutaneous pinning of elderly unstable distal radius fractures

## Abstract

**Background:**

Based on debatable recommendations of using bone substitutes for filling of metaphyseal void in elderly unstable distal radius fractures; this study investigated the following question “Do bone substitutes effectively contribute to postoperative stability of k-wire fixation construct and accelerate healing in elderly unstable distal radius fractures?”.

**Methods:**

This prospective cohort study was conducted from October 2014 to April 2021. According to use of bone substitutes, 40 patients of elderly unstable distal radius fractures were alternately allocated into; group-(A) of bone substitutes-augmented percutaneous pinning (19 patients); and group-(B) of non-augmented percutaneous pinning (21 patients). Groups were compared for preoperative patients’ demographics and postoperative ROM, Quick-DASH and Mayo Wrist scores, radiographic parameters (palmar tilt, radial height and inclination, ulnar variance and intra-articular step-off) and duration until radiographic fracture healing.

**Results:**

Statistically, augmented and non-augmented groups were matched in terms of patients’ demographics (mean age; 58.7 vs. 62.0 years respectively, *P*-value = 0.25). All included fractures have healed with insignificantly longer duration in augmented group (7.1 vs. 6.8 weeks, *P*-value = 0.26). At 12-week postoperative evaluation, radiographic parameters of both groups were comparably well-maintained except for intra-articular step-off which showed significantly less secondary displacement in augmented group (0.1 vs. 0.4 mm, *P*-value = 0.01). There were insignificant differences in 6-month postoperative ROM, and Quick-DASH and Mayo Wrist scores.

**Conclusion:**

Compared to its bone substitutes-augmented counterpart; non-augmented percutaneous pinning of elderly unstable distal radius fractures can offer advantages of comparable healing rates and functional and radiographic outcomes, less-invasive approach, shorter operative time and lower cost.

**Level of evidence:**

III

## Highlights

1. When used to augment percutaneous pinning of elderly unstable distal radius fractures, bone substitutes did not significantly offer additional postoperative mechanical stability of fixation construct.
2. When used to augment percutaneous pinning of elderly unstable distal radius fractures, bone substitutes did not significantly accelerate fracture healing.
3. Compared to bone substitutes-augmented pinning; stand-alone percutaneous pinning of elderly unstable distal radius fractures can achieve comparable healing rates and satisfactory functional and radiographic outcomes; and in addition, less-invasive approach, shorter operative time and lower cost.

## Introduction

In current orthopedic practice, bone-substitutes augmentation of fixation methods of elderly unstable distal radius fractures (DRF) represents an attractive management option. This attraction might partly stem from the reported mechanical advantages of these substitutes; i.e., filling of the metaphyseal void encountered in DRF is to offer immediate structural support and reinforced primary stability of the fixation construct of DRF [1–7].

Furthermore, enhancement of local biology for fracture healing (i.e., via osteoconductive and in some instances osteoinductive properties of bone substitutes) adds more drive for the orthopedic surgeons to exercise this option. It is needless to mention that use of bone substitutes avoids donor site morbidity of iliac bone auto-grafting and risk of antigenicity and disease transmission of bone allograft [1–7].

However, this option of bone substitutes-augmented fixation of DRF has its demerits of higher cost, longer operative time and possible intra-articular escape of bone substitutes. Besides, literature reported no clear evidence of superior functional and radiographic outcomes of this option compared with non-augmented fixation [4–9].

Based on this debate, the current study was conducted in order to investigate the following questions; (1) *“Do bone substitutes contribute effectively to postoperative stability of K-wire fixation construct of elderly unstable distal radius fractures?”* and (2) *“Do bone substitutes accelerate healing of elderly unstable distal radius fractures?”*

## Patients and methods

The currently-reported prospective cohort study (conducted between October 2014 and April 2021) was approved by the Institutional Committee of Scientific Research and Ethics. Initially, 57 patients were enrolled according to the following criteria; (1) patients of age older than 55 years and with unstable AO/OTA type-C distal radius fractures. Criteria of fracture instability was at least one of the following X-ray findings; >50% dorsal metaphyseal comminution, palmar metaphyseal comminution, >20^O^ initial dorsal tilt of the distal fracture fragment, >1cm initial radial translation of the distal fracture fragment, >5mm initial radial shortening, intra-articular fracture extension; or severe osteoporosis. Other criteria of enrollment were (2) sound contralateral wrist; (3) signed written well-informed patient consent; and (4) availability for a minimum of 6-month postoperative follow-up.

**Fig. 1:**
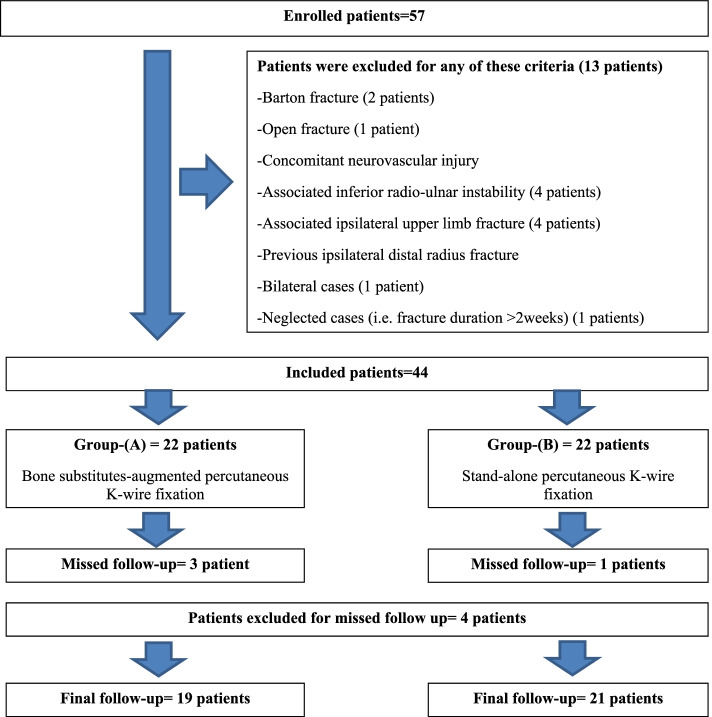
Illustrates a flowchart of patients enrolled in the current study

However, 13 patients were excluded for one or more of the following criteria; (1) Barton fracture (2 patients); (2) open fracture (1 patient); (3) concomitant neurovascular injury; (4) associated inferior radio-ulnar instability (4 patients); (5) associated ipsilateral upper limb fracture (2 patients of distal ulnar diaphyseal/metaphyseal fracture, 1 patient of humeral shaft fracture and 1 patient of comminuted fracture of the radial head); (6) previous ipsilateral distal radius fracture; (7) bilateral cases (1 patient); and finally, (8) neglected cases (i.e. fracture duration >2weeks) (1 patients). Figure 1 demonstrates flowchart of patients enrolled in the current study.

Accordingly, the current study included 44 patients of DRF managed by closed reduction under anesthesia and percutaneous K-wire fixation. According to bone substitutes augmentation of the K-wire fixation construct; included patients were alternately allocated into two groups at a ratio of (1:1); i.e. patients of odd numbers of inclusion array (e.g., 1, 3, 5….etc.) were allocated into group-(A) of bone substitutes-augmented percutaneous K-wire fixation; meanwhile, patients of even numbers of inclusion array (e.g., 2, 4, 6,…..etc.) were allocated in group-(B) of non-augmented percutaneous K-wire fixation. This reciprocal allocation of included patients into the studied groups was strictly followed regardless of severity of metaphyseal/dorsal comminution of DRF.

Likewise, 4 patients missed postoperative follow-up; hence, data of 40 patients; i.e., 19 patients in group-(A) and 21 patients in group-(B) were available for statistical analysis of the investigated outcomes.

Preoperatively, patients were subjected to detailed history taking (i.e. age, mode of trauma, duration elapsed since injury and medical co-morbidities). In addition, patients were examined for concomitant extra-skeletal injuries, associated fractures, ipsilateral neuro-vascular compromise, and local soft tissue injury. All included patients were radio-graphically evaluated by antero-posterior and lateral X-ray views of the affected distal forearm and wrist.

### Operative details

Following (general/regional) anesthesia, prophylactic antibiotic administration and supine positioning of the patient, closed reduction of DRF was achieved under imaging intensifier via traction/counter-traction technique with special attention dedicated to restoration of radiographic parameters of accepted reduction. In some instances, reduction was achieved by joy-stick/intra-focal Kapandji technique.

**Fig. 2:**
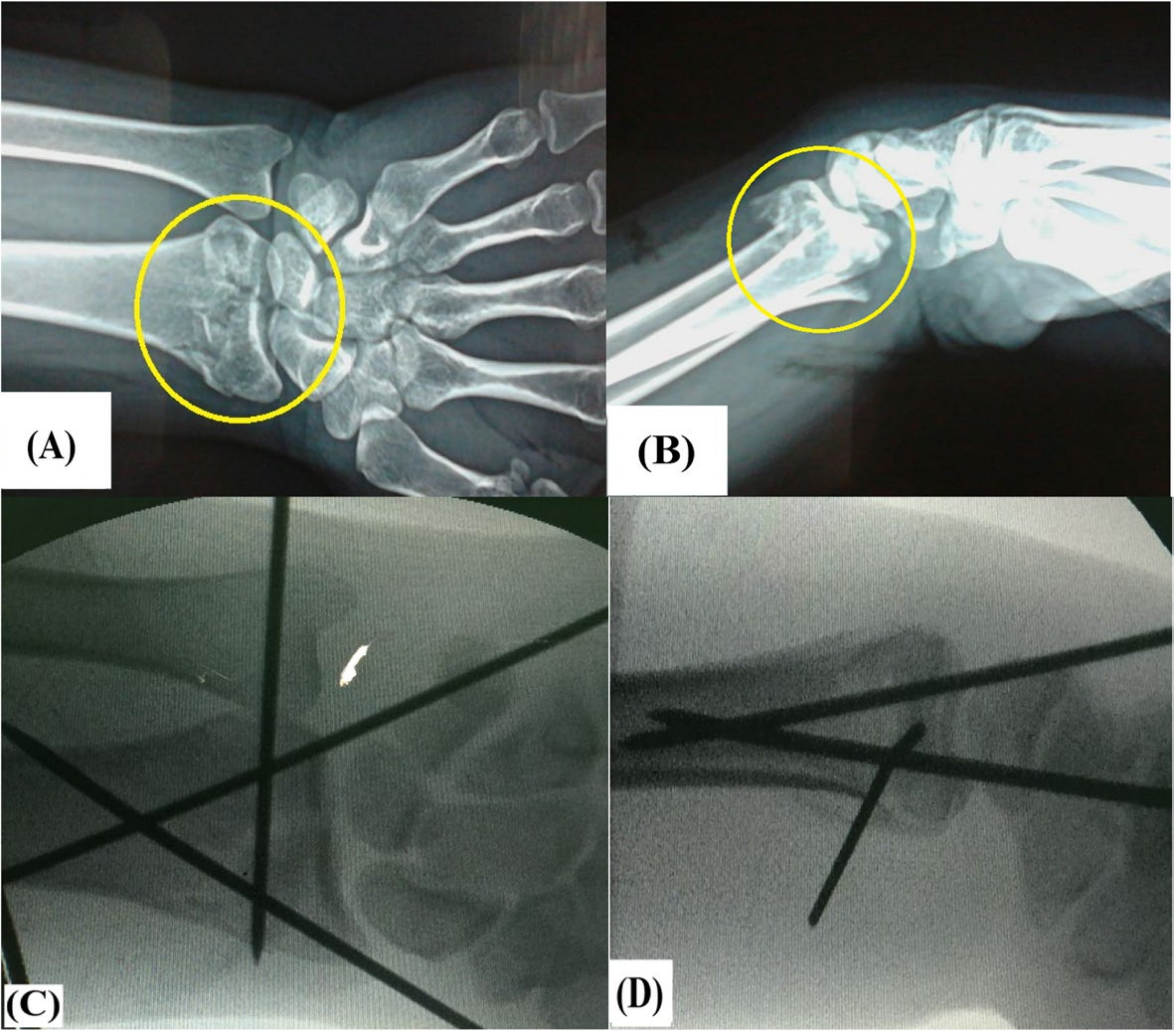
Demonstrate unstable intra-articular distal radius fracture in 56 years old female patient; **A** & **B** preoperative antero-posterior and lateral X-ray views of the fracture (marked in yellow circle); **C** & **D** intra-operative antero-posterior and lateral imaging intensifier views of triangular configuration of percutaneous K-wire fixation following closed reduction of the fracture

Then, reduced fracture was stabilized using percutaneous pinning technique with triangular configuration of 3 (1.5-2mm) K-wires. Firstly, 2 K-wires were placed in cis-cross fashion within distal radial metaphysis (i.e., 1^st^ pin was inserted through the radial styloid and the second pin was inserted through the dorso-ulnar fragment). Following this step, stability of inferior radio-ulnar joint (IRUJ) was evaluated. In patients with stable IRUJ, the 3^rd^ pin was placed either through the radial styloid into subchondral bone of the distal radius almost parallel to the radiocarpal joint till bi-cortical engagement of the distal ulna (for additional stability of the fixation reconstruct); or vice versa. Meanwhile, in patients with unstable IRUJ; this joint was reduced and stabilized using a K-wire; however, those patients were excluded from the study. Figure 2A, B, C and D demonstrate preoperative antero-posterior and lateral X-ray views of unstable intra-articular DRF and antero-posterior and lateral imaging intensifier views of triangular configuration of percutaneous K-wire fixation of reduced DRF.

**Fig. 3:**
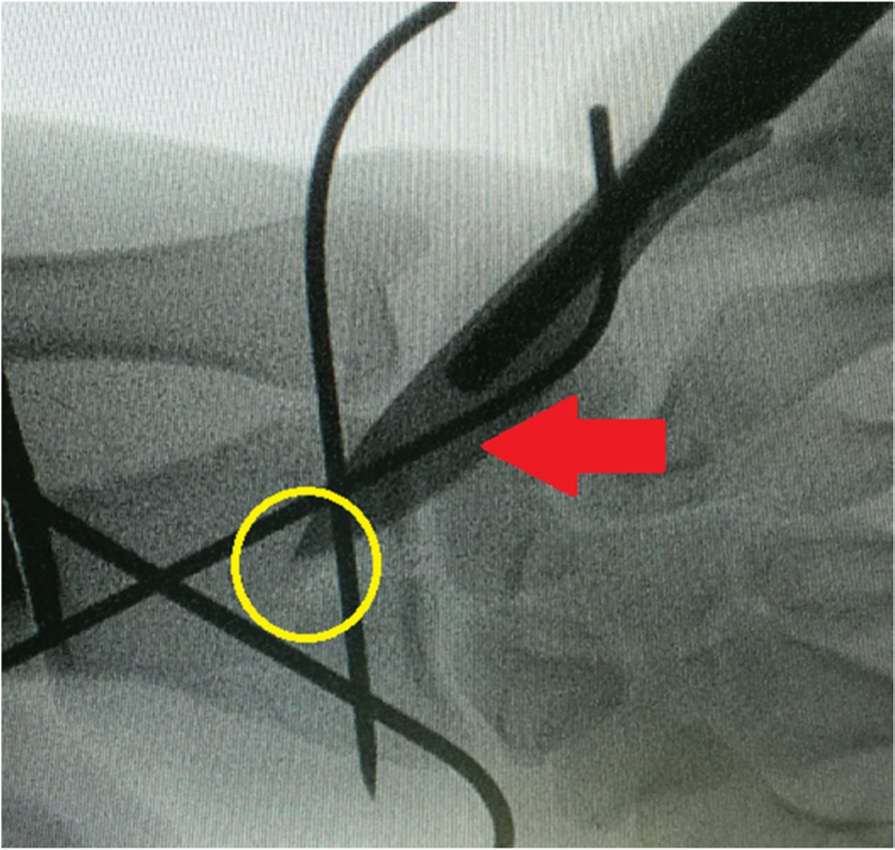
Demonstrates intra-operative planning for dorsal approach of the distal radius; i.e., 2.5 cm skin incision was planned over dorsal aspect of the distal radius under imaging intensifier using (red arrow-marked) skin scalpel centered over the metaphyseal comminution (marked in yellow circle)

In bone substitutes-augmented group, 2.5cm skin incision was performed over dorsal aspect of the distal radius. In order to be centered over the metaphyseal comminution; this incision was planned under imaging intensifier. Figure 3 demonstrates planning of skin incision over dorsal aspect of the distal radius under imaging intensifier.

**Fig. 4:**
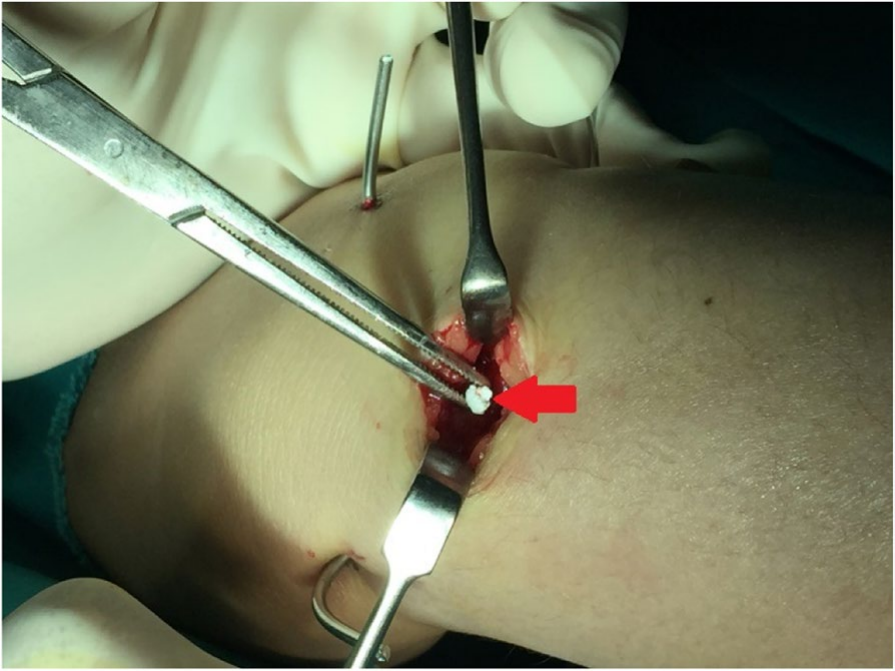
Demonstrates impaction of the (red arrow-marked) bone substitutes within fracture void of distal radial metaphysis

Afterwards, sub-cutaneous tissue was dissected in line with skin incision, extensor tendons were retracted and protected, and fracture defect was identified. Through this dorsal cortical defect; the fracture site was debrided and irrigated; and also, residual intra-articular step (if any) was reduced under imaging intensifier using a blunt instrument (i.e., no dorsal wrist capsulotomy was performed for direct intra-articular visualization). Then, the fracture void was filled using thoroughly-impacted average volume of 5cc of granular hydroxyapatite-β tricalcium-phosphate bone substitutes (Poly Bone^R^ Granule, Biomatlante, 44360 Vigneux-de-Bretagne, France). Figure 4 demonstrates impaction of bone substitutes within metaphyseal void of the distal radius fracture.

**Fig. 5:**
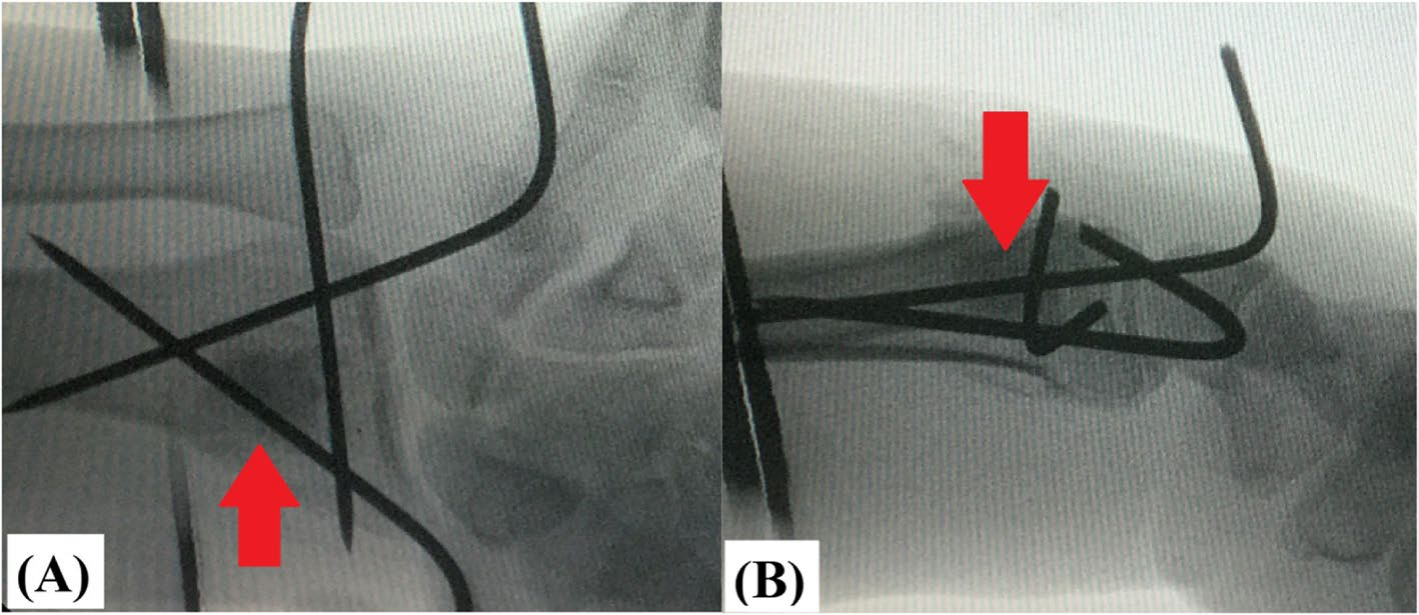
Demonstrates completed technique of (red arrow-marked) bone substitutes-augmented percutaneous K-wire fixation of reduced distal radius fracture; **A** antero-posterior; and **B** lateral imaging intensifier views

Thereafter, radiographic evaluation (under imaging intensifier) of the final construct was routinely performed to ascertain adequate defect filling, exclude intra-articular/soft tissues deposits of bone substitutes and ensure stable accepted fracture reduction/fixation under dynamic testing. Figure 5A and B demonstrate antero-posterior and lateral imaging intensifier views of completed technique of bone substitutes-augmented percutaneous K-wire fixation of reduced DRF.

In both groups, distal radial and ulnar pulsations and capillary filling were checked prior to fracture splinting.

All patients included in the current study were operated by or under direct supervision of the author.

### Postoperative rehabilitation protocol

In both groups, operated forearm was splinted with wrist in neutral position for 6weeks. Measures guarding against complex regional pain syndrome (CRPS) were strictly implemented including strong analgesia (e.g. NSAIDs), arm elevation and ice packs for the 1^st^ postoperative 48 hours. Pin care and wound dressing were performed on a weekly basis. Active range of motion (ROM) exercises of the fingers were encouraged throughout splintage duration. By end of the 6^th^ postoperative week, splint was discarded and K-wires were removed in out-patient clinic in order to allow the patient to resume light daily-living activities and to initiate passive (stretching) and active (strengthening) ROM exercises of the wrist for another 6 weeks. Return to work was allowed by an average of 2.5 postoperative months.

### Outcome measurements

In the current study, patients were closely followed-up at 1^st^, 2^nd^, 6^th^, 8^th^, 12^th^ and 24^th^ postoperative weeks for assessment of the clinical parameters (e.g. pain/tenderness over the distal radius, goniometer-based measurement of active ROM of the wrist, return to work/sports activity, and complications); and also for rating of the functional outcomes using Quick-Disabilities of the Arm, Shoulder and Hand (DASH) and Mayo Wrist scoring systems.

Meanwhile, radiographic evaluation (i.e., using antero-posterior and lateral X-ray views) included measurement of the following parameters; palmar tilt (PT), radial height (RH), radial inclination (RI), ulnar variance (UV) and intra-articular step-off (IN-S). Radiographic evaluation also included postoperative duration until radiographic fracture healing; the latter was defined as healing of 3 out of four cortices of the managed DRF.

Differences in measurements (i.e. secondary displacement/collapse) of radiographic parameters between immediate and 12-week postoperative X-ray views; and in addition, 6-month postoperative clinical and functional data (as assessed by the surgeon/author) were used for statistical analysis.

### Statistical analysis

Data were collected, tabulated and statistically-analyzed using IBM Statistical Package of Social Science (SPSS) software, version 20 (IBM Co., Armonk, NY, USA). Based on review of past literature, sample size calculation of study power of 80% at confidence interval of 95% rendered 30 patients allocated into 2 groups; e.g., exposed versus non-exposed with 10% fall-out. For data comparison, *t-*test, Chi-square test and Mann-Whitney test were used; with *P-*value of <.05 for significance. Statistical analysis was performed by an independent statistician.

## Results

As regards patients’ demographics, studied groups were statistically matched. The most common mode of trauma was falling on outstretched hand; i.e., 17 (89.5%) and 20 (95.2%) patients in group-(A) and group-(B) respectively. Table 1 demonstrates a summary of patients’ demographics of the studied groups.

**Table 1:** Demonstrates a summary of patients’ demographics of the studied groups

Characteristics	Studied Groups				Test Statistic	*P*-value
	**Augmented** **Group** **(No. = 19 patients)**		**Non-augmented** **Group** **(No. = 21 patients)**			
	Mean ± SD		Mean ± SD			
**Age (in years)**	58.78 ± 9.39 Range = 56–73		62.0 ± 8.27 Range = 55–71		***t*-test = 1.14**	0.25
**Gender**						
Male	5	26.3%	6	28.6%	**Chi-square test** **χ**^**2**^** = 0.02**	0.87
Female	14	73.7%	15	71.4%		
**Hand Dominance**						
Dominant	11	57.9%	15	71.4%	**Chi-square test** **χ**^**2**^** = 0.80**	0.37
Non-dominant	8	42.1%	6	28.6%		
**AO-Classification**						
Type-C1	7	36.8%	8	38.1%	**Chi-square test** **χ**^**2**^** = 0.66**	0.95
Type-C2	5	26.4%	7	33.3%		
Type-C3	7	36.8%	6	28.6%		

**Fig. 6:**
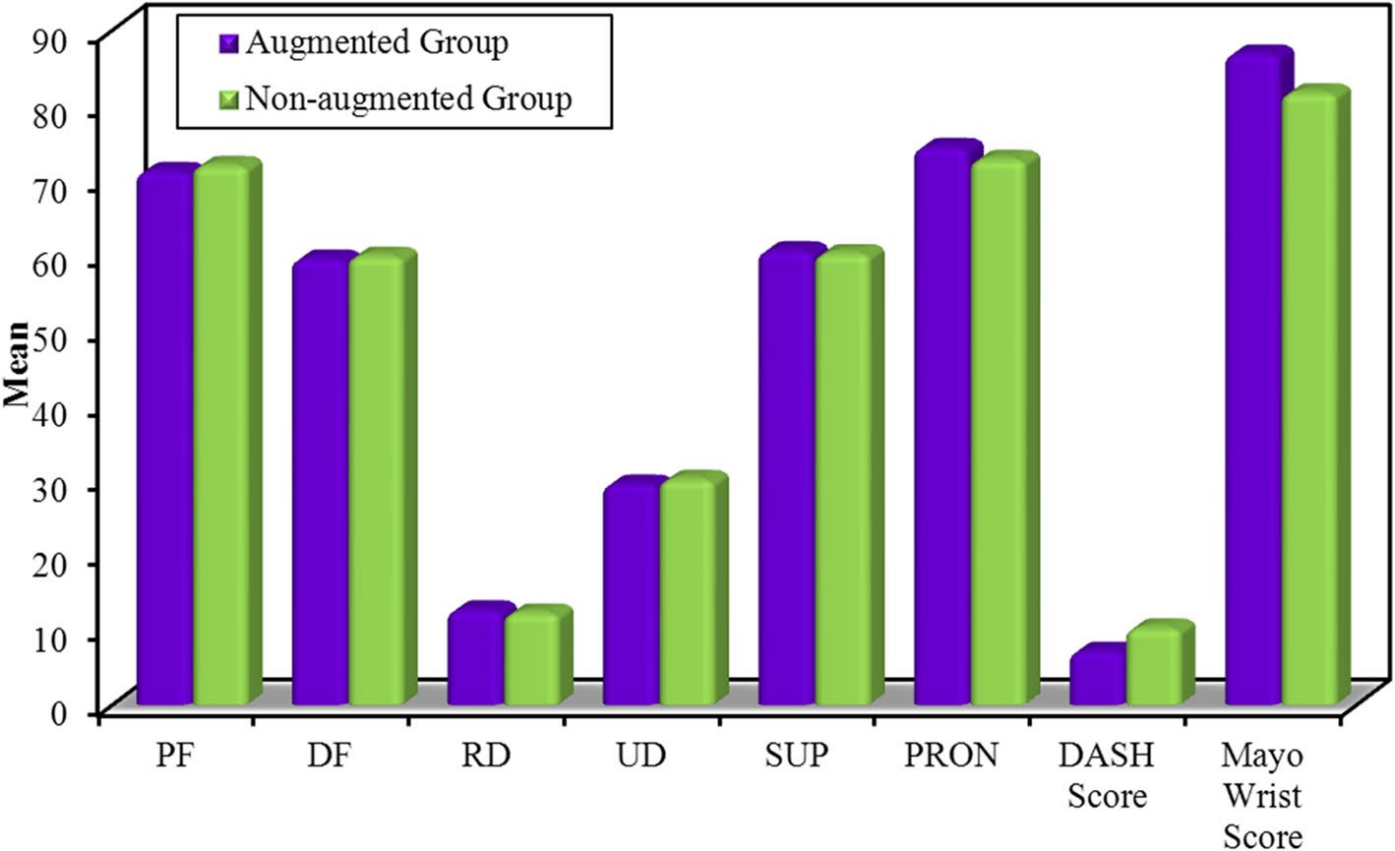
Illustrates postoperative outcome measurements of range of motion and Quick-DASH and Mayo Wrist scoring systems of the studied groups; PF, Palmar Flexion; DF, Dorsal Flexion; RD, Radial Deviation; UD, Ulnar Deviation; SUP, Supination; PRON, Pronation

At 6-month postoperative evaluation, there were insignificant differences between the studied groups in terms of active ROM and Quick-DASH and Mayo wrist scoring systems. Postoperative outcome measurements of ROM and Quick-DASH and Mayo Wrist scoring systems of the studied groups are summarized in Table 2 and illustrated in Fig. 6.

**Table 2:** Summarizes postoperative outcome measurements of range of motion and Quick-DASH and Mayo Wrist scoring systems of the studied groups

	**Studied Groups**		**Test Statistic**	***P*-value**
	**Augmented** **Group** **(No. = 19 patients)**	**Non-augmented** **Group** **(No. = 21 patients)**		
	Mean ± SD	Mean ± SD		
**Palmar Flexion** **(in degrees)**	71.15 ± 5.63 Range = 58–80	71.95 ± 5.87 Range = 58–81	***t*-test = 0.43**	0.66
**Dorsal Flexion** **(in degrees)**	59.47 ± 5.49 Range = 44–67	59.85 ± 4.55 Range = 51–69	***t*-test = 0.24**	0.81
**Radial Deviation** **(in degrees)**	12.68 ± 2.33 Range = 8–16	12.28 ± 1.95 Range = 8–15	***t*-test = 0.58**	0.56
**Ulnar Deviation** **(in degrees)**	29.47 ± 4.53 Range = 21–36	30.09 ± 4.61 Range = 23–38	***t*-test = 0.42**	0.67
**Supination** **(in degrees)**	60.57 ± 5.39 Range = 49–68	60.19 ± 5.05 Range = 51–68	***t*-test = 0.23**	0.81
**Pronation** **(in degrees)**	74.36 ± 4.85 Range = 60–81	72.76 ± 5.44 Range = 64–82	***t*-test = 0.98**	0.33
**Quick-DASH Score**	7.17 ± 11.11 Range = 0–50	10.28 ± 11.62 Range = 2.3–45.5	**Mann–Whitney test = ** **1.57**	0.11
**Mayo Wrist Score**	86.84 ± 17.65 Range = 30–100	81.66 ± 19.32 Range = 25–95	**Mann–Whitney test = ** **1.48**	0.13

**Fig. 7:**
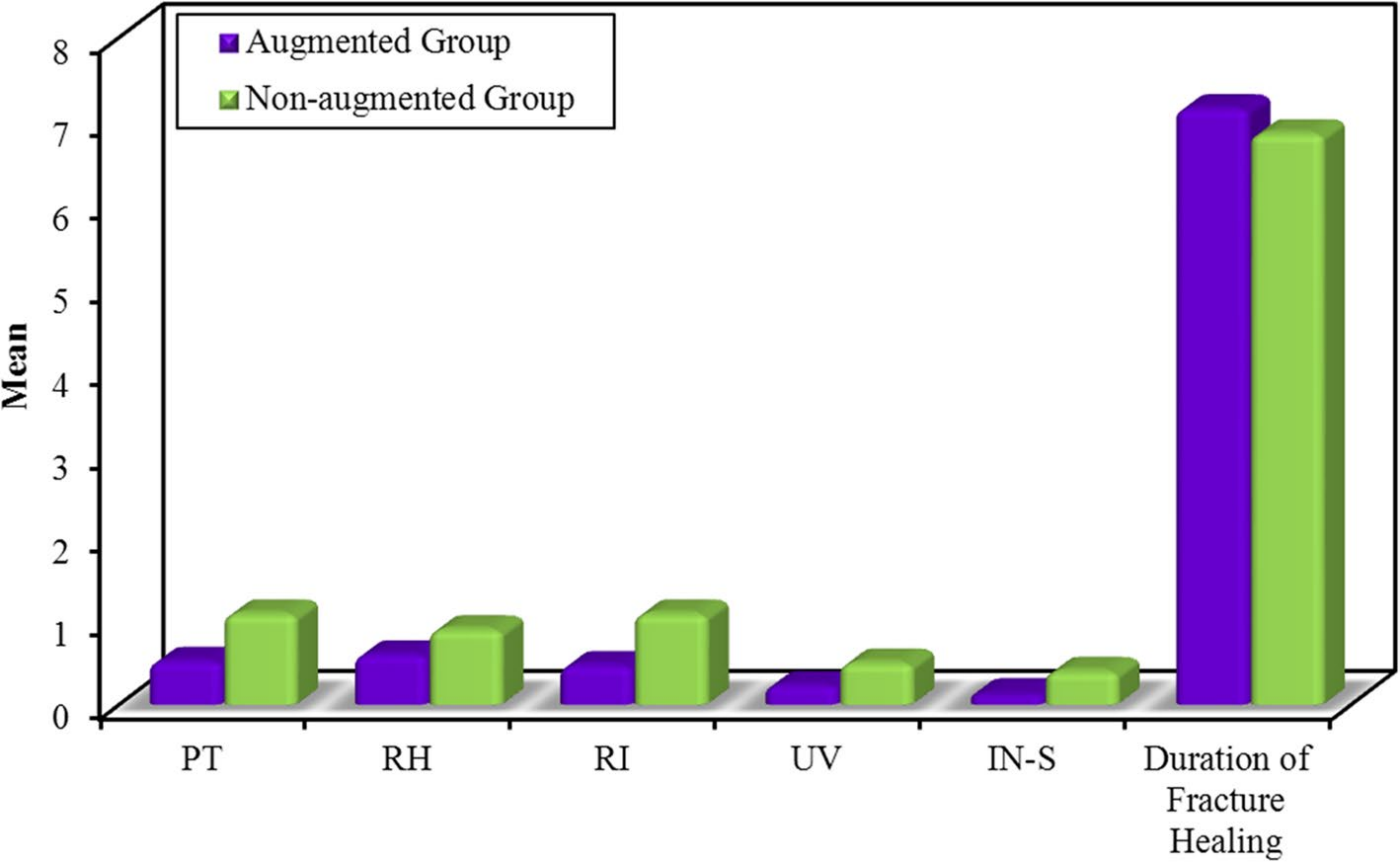
Illustrates postoperative outcome measurements of secondary displacement in the radiographic parameters and postoperative duration until radiographic fracture healing of the studied groups; PT, Palmar Tilt; RH, Radial Height; RI, Radial Inclination; UV, Ulnar Variance; IN-S, Intra-articular Step-off

With regard to 12-week postoperative radiographic outcomes, statistical analysis revealed insignificantly less secondary displacement of the radiographic parameters (except for intra-articular step-off) in the augmented group compared with the non-augmented one. Otherwise, intra-articular step-off showed significantly less secondary displacement in the augmented group (0.1 vs. 0.4 mm, *P-value*=0.01). Meanwhile, postoperative duration until radiographic fracture healing was insignificantly longer in the augmented group (7.1 vs. 6.8 weeks, *P-value*=0.2). Postoperative outcome measurements of secondary displacement of the radiographic parameters and postoperative duration until radiographic fracture healing of the studied groups are summarized in Table 3 and illustrated in Fig. 7.

**Table 3:** Summarizes postoperative outcome measurements of secondary displacement of the radiographic parameters and postoperative duration until radiographic fracture healing of the studied groups

	**Differences between Immediate and 12-week Postoperative X-ray**		**Test Statistic**	***P*-value**
	**Augmented** **Group** **(No. = 19 patients)**	**Non-augmented Group** **(No. = 21 patients)**		
	Mean ± SD	Mean ± SD		
**Palmar Tilt** **(in Degrees)**	0.52 ± 0.77 Range = 0–2	1.09 ± 1.22 Range = 0–4	**Mann–Whitney test = ** 1.60	0.10
**Radial Height** **(in mm)**	0.60 ± 0.84 Range = 0–3	0.90 ± 1.22 Range = 0–4	**Mann–Whitney test = ** 0.40	0.68
**Radial Inclination** **(in mm)**	0.47 ± 0.61 Range = 0–2	1.09 ± 1.17 Range = 0–4	**Mann–Whitney test = ** 1.71	0.08
**Ulnar Variance** **(in mm)**	0.23 ± 0.51 Range = 0–2	0.50 ± 0.60 Range = 0–2	**Mann–Whitney test = ** 1.92	0.05
**Intra-articular Step-off** **(in mm)**	0.13 ± 0.28 Range = 0–1	0.40 ± 0.40 Range = 0–1	**Mann–Whitney test = ** 2.35	0.01*
**Duration of Fracture Healing** **(in Weeks)**	7.15 ± 0.76 Range = 6–8	6.85 ± 0.91 Range = 6–8	**Mann–Whitney test = ** 1.12	0.26

^*^Significant

In the current study, the most common postoperative complication was pin tract infection diagnosed in 15 (37.5%) patients. Postoperative complications of the studied groups are summarized in Table 4.

**Table 4:** Summarizes postoperative complications of the studied groups

Complications	Augmented Group (No. = 19 patients)	Non-augmented Group (No. = 21 patients)
Pin tract infection	7 (36.8%)	8 (38.1%)
Pin loosening & early pin removal	1 (5.3%)	3 (14.3%)
Complex regional pain syndrome	3 (15.8%)	4 (19%)
Mild ulnar-sided wrist pain on exertion	2 (10.5%)	3 (14.3%)

## Discussion

When reviewed, literature reveals no consensus on management algorithm of unstable DRF in elderly populations. Different options of surgical approaches, fixation methods and graft materials have been described reporting debatable outcomes [1–7].

Of these controversial options is augmentation of DRF fixation with synthetic bone substitutes. Indeed, the latter option has gained increasing popularity among orthopedic surgeons because of its potential mechanical and biological advantages. However, there is still no robust evidence of superiority of bone substitutes-augmented fixation of DRF compared with its non-augmented counterpart [4–6].

Based on this debate, the current study was conducted to investigate the following questions; (1) *“Do bone substitutes contribute effectively to postoperative stability of K-wire fixation construct of elderly unstable distal radius fractures?”* and *(2) “Do bone substitutes accelerate healing of elderly unstable distal radius fractures?”*; assuming that bone substitutes-augmented percutaneous pinning is to show significantly higher primary stability of the fixation construct and shorter duration for fracture healing.

### Outcomes of the study

#### “Do bone substitutes contribute effectively to postoperative stability of fixation construct of elderly unstable distal radius fractures?”

The most important finding of the present study was that use of bone substitutes for augmentation of percutaneous pinning of elderly unstable DRF didn’t significantly add to mechanical stability of the fixation construct; this finding can be clued from statistically-comparable secondary displacement of postoperative radiographic parameters between studied groups. Figs. 

 8A, B, C, D, E, F and 9A, B, C, D, E, F demonstrate insignificant secondary displacement of postoperative radiographic parameters of augmented and non-augmented percutaneous pinning of DRF respectively.

**Fig. 8:**
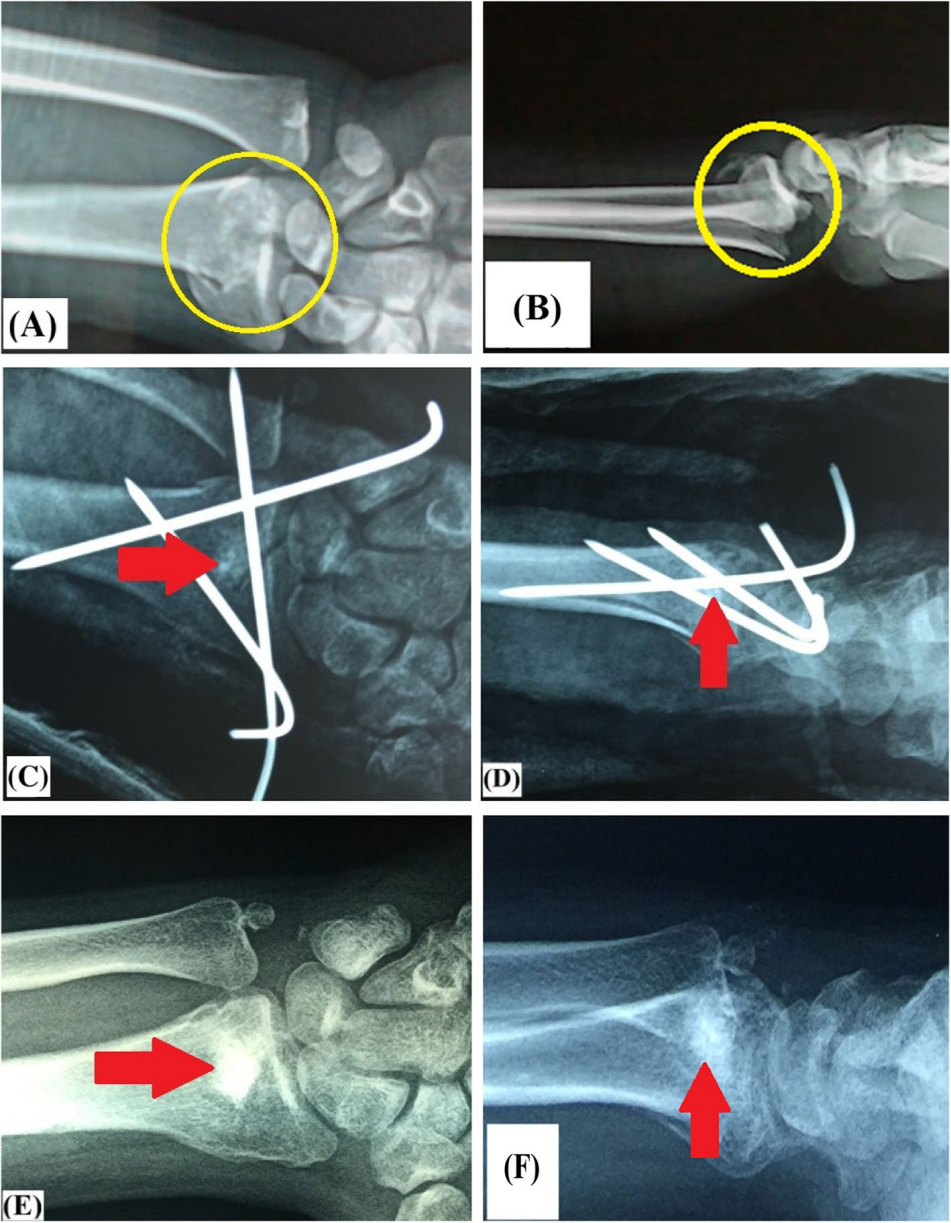
Demonstrate insignificant secondary displacement of postoperative radiographic parameters of unstable intra-articular distal radius fracture in 57-year old male patient managed by bone substitutes-augmented percutaneous pinning; **A** & **B** preoperative antero-posterior and lateral X-ray views of the fracture (marked in yellow circle); **C** & **D** immediate postoperative antero-posterior and lateral X-ray views of (red arrow-marked) bone substitutes-augmented percutaneous pinning of the fracture; **E** & **F** 12-week postoperative antero-posterior and lateral X-ray views of (red arrow-marked) bone substitutes-augmented percutaneous pinning of the fracture

**Fig. 9:**
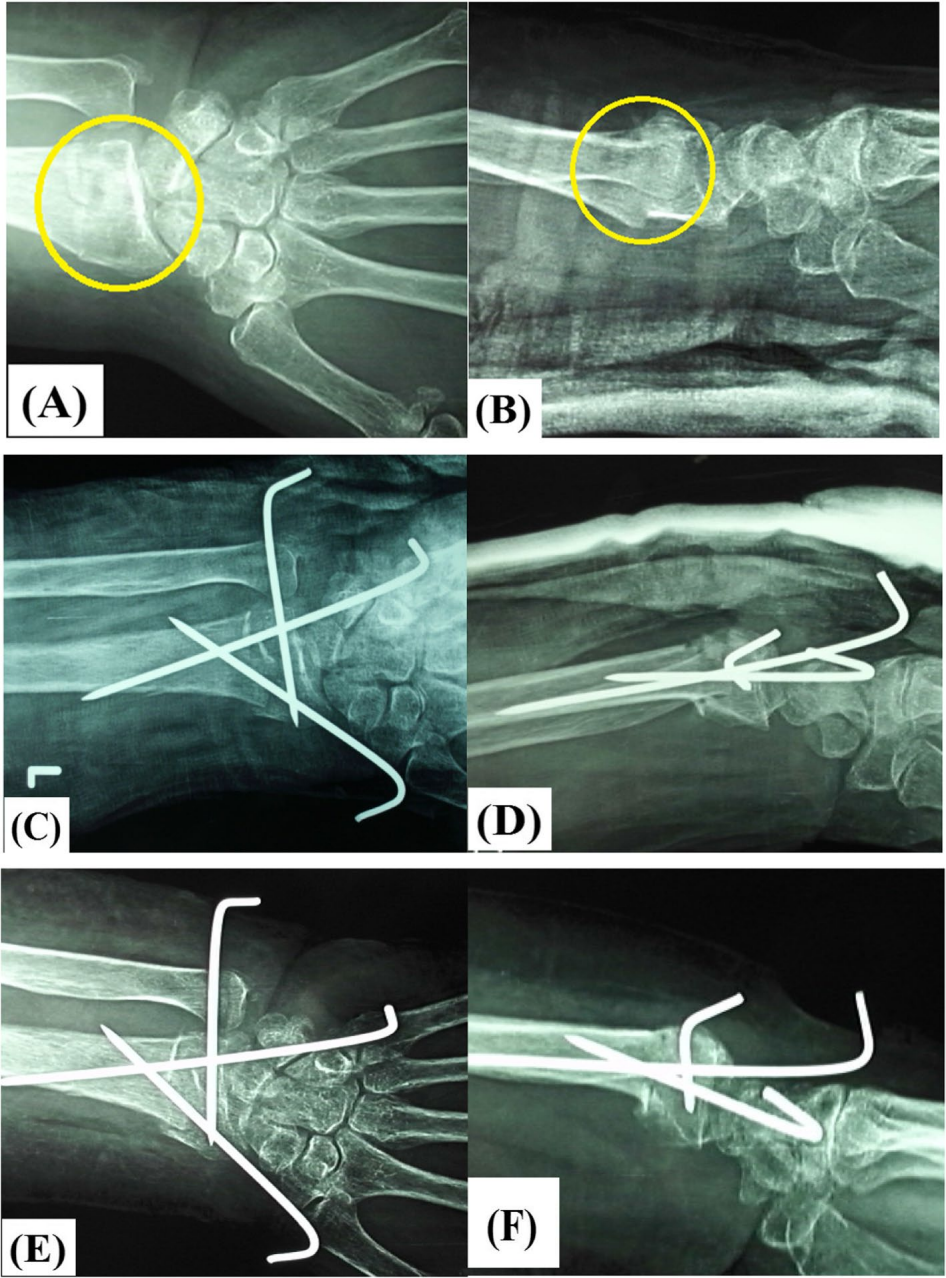
demonstrate insignificant secondary displacement of postoperative radiographic parameters of unstable intra-articular distal radius fracture in 64-year old female patient managed by stand-alone percutaneous pinning; **A** & **B** preoperative antero-posterior and lateral X-ray views of the fracture (marked in yellow circle); **C** & **D** immediate postoperative antero-posterior and lateral X-ray views of the fracture; **E** & **F** 6-week postoperative antero-posterior and lateral X-ray views of the fracture

The preceding finding is also reported by other authors utilizing a different fixation method of DRF; i.e. plating. In a prospective randomized trial of dorsally-plated comminuted elderly DRF allocated into two groups (i.e. bone substitute-augmented group of 19 patients and non-augmented group of 20 patients); Jakubietz et al showed 1-year postoperative insignificant differences in functional scores and radiographic parameters between groups with better ROM and grip strength in non-augmented group; pointing out that no clear mechanical advantages of added granular bone substitutes could be concluded [4].

In a cohort study investigating role of bone substitutes in maintaining DRF reduction through comparing radiographic parameters of non-augmented versus hydroxyapatite bone substitutes-augmented volar plating of elderly unstable DRF; Goto et al stated that there were insignificant differences (except for ulnar variance) between groups [10].

On the other hand, currently-reported insignificant mechanical advantages of bone substitutes might be contradicted by recommendation of Hedge et al who advocated bone substitutes-augmented percutaneous pinning in a prospective case series (i.e. no control group) of 27 elderly DRF; reporting insignificant marginal changes in radiographic parameters at 16-week postoperative follow-up [5].

In addition, another contradiction might be inferred from a cadaveric study (including 28 fresh-frozen cadavers) of Kainz et al who elaborated significantly-improved mechanical properties (e.g., decreased displacement and increased stiffness under cyclic loading) of injectable hydroxyapatite bone substitutes-augmented volar plate fixation of simulated AO/OTA type-A3 distal radius fractures compared with non-augmented plating [11].

In the current study, the sole significant difference in radiographic parameters between the studied groups (i.e., significantly less secondary displacement of intra-articular step-off in the augmented group) might favor presumed advantage of structural support of the bone substitutes in management of DRF. However, this radiographic difference didn’t result in significant clinical differences (i.e., in terms of ROM and functional scoring systems) between the studied groups. Up to date, correlations of postoperative radiographic parameters of DRF with long-term functional outcomes and post-traumatic osteoarthritis have not been well established [1, 4, 12–15].

In a retrospective review of 51 patients of unstable intra-articular DRF managed with volar locked plating; Dario et al stated that radiographic parameters of significant correlation with 3-year postoperative ROM and functional outcomes were ulnar variance and volar tilt rather than intra-articular step-off [12].

Meanwhile, in a randomized controlled trial of 73 elderly patients (age: ≥ 65 years) of unstable DRF assigned between a group of volar plating (36 patients) and a second group of closed reduction and casting (37 patients); Arora et al reported that by 6^th^ and 12^th^ postoperative months, radiographic parameters were significantly better in plated fractures; however, this better radiographic reduction did not result in significantly superior ROM and functional outcomes compared with conservatively- managed fractures [1].

In systematic review of 21 studies of 5 different management modalities of elderly unstable DRF, Diaz et al concluded that despite cast immobilization following closed reduction showed the worst radiographic parameters; however, it had indifferent functional outcomes and the lowest complication rate compared with operative options (e.g., percutaneous pinning, volar locked plating and birding and non-bridging external fixators) [15].

#### “Do bone substitutes accelerate healing of elderly unstable distal radius fractures?”

Another important finding of the current study was that bone-substitutes augmentation of elderly unstable DRF pinning might insignificantly delay fracture healing. This finding could be explained by loss of fracture hematoma during surgical dissection for impaction of the bone substitutes. In addition, healing of all fractures in non-augmented group might negate biological necessity of the bone substitutes for healing enhancement in DRF.

Likewise, reviewed literature heralds minimal concerns about non-union of DRF. In a prospective case series of 60 patients of DRF (i.e., AO-types A3 and C3) managed with non-augmented volar locked plating i.e., without bone graft or substitutes; Garcés-Zarzalejo et al demonstrated healing of all included fractures without significant reduction loss; stating that bone grafts/substitutes were not mandatory in management of unstable DRF [6].

### Technical considerations

#### Bone substitutes

In spite of controversial superiority of block form of the bone substitutes in preventing secondary displacement of DRF; granular form was of choice in the present study because of its easier application within the metaphyseal void.

Besides, impaction of the bone substitutes was performed via the dorsal approach because it enables the surgeon to directly tackle the void through the dorsal metaphyseal comminution (i.e., without creating a cortical window); and also, this approach facilitates direct visualization (when needed) of articular surface the distal radius through dorsal capsulotomy.

In the current study, average estimated cost of used bone substitutes was 48$ /case which might significantly add to the increasing economic burden of DRF management especially in the light of common incidence of DRF (i.e., the second most common elderly skeletal fracture; accounting for 18% of all skeletal fractures); and in addition, current tendency of longer life expectancy and relatively higher level of activity among aging population than in past decades [15–17].

#### Method of fixation

In the present study, percutaneous pinning was selected over other methods of fixation (e.g., volar plating and external fixator) because of patient related-factors (e.g., elderly age group with relatively higher prevalence of co-morbidities necessitating short operative time; and with relatively lower functional demands), significantly lower cost, minimally-invasive approach, and no need for reoperation for hardware removal [8, 18, 19]. In a cost-effective study, Franer et al reported that compared with percutaneous pinning; volar plating had significantly higher charges, payments and complication rate (e.g. median nerve irritation/entrapment and tendon rupture) [8].

Another factor was comparable reported functional and radiographic outcomes of commonly-used methods of fixation [2, 3, 7, 9, 18, 20]. In a randomized controlled study of 461 patients of DRF managed in 18 different trauma centers across United Kingdom, Costa et al reported no differences in functional outcomes of volar locked plating versus K-wire fixation; however, the later was cheaper and quicker to perform [2]. In a meta-analysis of 7 randomized controlled studies of 875 DRF, Chaudhry et al concluded that volar plating showed marginal superiority over percutaneous pinning in terms of ROM and functional scores mainly at early (i.e., 3-month) postoperative follow-up; however, radiographic parameters were statistically comparable at both 3- and 12-month postoperative evaluation [7]. Zong et al stated similar conclusion in an independent meta-analysis [20]. In a systematic review of 14 studies (10 of which were prospective randomized controlled trials) including 1306 patients of DRF, Franceschi et al demonstrated that both percutaneous pinning and volar locked plating achieved satisfactory functional and radiographic outcomes; and moreover, there was no clear superiority of either of the examined fixation methods [9].

#### Ulnar styloid

A point worth mentioning is that in the current study; no operative intervention was dedicated to associated fracture of the ulnar styloid as these fractures frequently heal and non-united fractures of the ulnar styloid are usually asymptomatic [21]. In addition, patients with concurrent IRUJ instability following DRF fixation were initially excluded in order to augment internal validity of the current study.

#### Arthroscopic approach

Otherwise, recent techniques of arthroscopic-assisted reduction and osteosynthesis of displaced intra-articular DRF are currently scrutinized. Rationales behind these techniques included persistence of intra-articular step-off (in 22% out of 249 DRF) even if reduction seemed to be achieved under fluoroscopy; exclusion of intra-articular screw penetration, associated carpal stability and concurrent injury of triangular fibro-cartilage complex; and furthermore, satisfactory functional and radiographic postoperative outcomes [22, 23].

Actually, in spite of well-developed wrist arthroscopic practice in the institution; arthroscopic-assisted reduction/fixation of DRF was not exercised in the current study due to factors related to insurance coverage, emergency operative theatre time management, patients’ co-morbidities, and expected high cost-effective ratio of the technique. The later drawback can be attributed to disadvantage of (1)-troublesome technical setup (e.g., alternating vertical traction application/release and maneuvering of imaging intensifier); and (2)-arthroscopy-related complications (e.g., fluid extravasation and neurological injury); both disadvantages surpass expected advantages of arthroscopic-assisted reduction/fixation.

### Complications

From another perspective, pin tract infection was diagnosed in 7 (36.8%) and 8 (38.1%) patients in the augmented and non-augmented groups respectively. Such infection was successfully managed by dressing and oral antibiotics except in 4 patients (i.e., 1 and 3 patients in the augmented and non-augmented groups respectively) in whom infection was further complicated by pin loosening necessitating earlier pin removal by an average of 4.7 postoperative weeks.

It goes without saying that precautions guarding against CRPS were strictly followed throughout the study; nevertheless, CRPS was early diagnosed in 7 patients; i.e., 3 (15.8%) and 4 (19%) patients in the augmented and non-augmented groups respectively. By final follow-up, this major complication has resolved aided by analgesics, anti-edematous medication, neurotonics, and aggressive ROM exercise.

No other major complications of neuro-vascular compromise, intra-articular escape of bone substitutes, wound and/or deep infection or tendon rupture could be reported.

### Consistency of the study

In this study, certain factors were considered to ensure consistency of study outcomes as; (1) prospective cohort study design; (2) strict adherence to inclusion and exclusion criteria to avoid bias related to inclusion of different fracture patterns or associated ipsilateral fractures; (3) matched studied groups; (4) standardized operative technique notably with regard to surgical approach, method/configuration of fixation and bone substitutes (i.e., all cases were performed by or under supervision of the author); (4) strict commitment to a standardized postoperative rehabilitation protocol; and finally, (5) use of postoperative secondary displacement of the radiographic parameters as an outcome for assessment of postoperative stability of the fixation construct.

It is essential to point out that assessment of radiographic parameters was carried out 6 weeks following K-wire removal (i.e., at a total of 12 postoperative weeks) to take into consideration possible post-removal secondary fracture displacement according to some reports and to ensure solid fracture healing prior to radiographic evaluation [2, 3, 9].

### Limitations

Undoubtedly, currently-reported study had its own limitations which included elderly patient groups (i.e., groups of low energy trauma and of relatively lower functional demands); small number of patients; exclusion of patients with concurrent IRUJ instability; use of granular form of bone substitutes (i.e., not solid blocks); technical variability in plain X-ray imaging protocol; and non-blinded assessment of outcome measurements.

In addition, radiographic outcomes were assessed by X-ray imaging solely without further CT evaluation. This is explained by widespread availability of X-ray imaging in common practice; and by radiation hazards and cost of CT imaging.

Moreover, duration of postoperative follow-up was relatively short; however, it was enough for assessment of primary outcome of the study (i.e., secondary displacement). On the other hand; incidence of post-traumatic osteoarthritis can’t be reported so that longer-term (e.g., 2-year) postoperative follow up studies are recommended.

Another limitation which might overshadow study outcomes is that it was carried out at a tertiary-level referral trauma center where surgeons are well-experienced in management of DRF.

## Conclusion

With respect to its bone substitutes-augmented counterpart; non-augmented percutaneous pinning of elderly unstable DRF is a less-invasive quicker technique which can achieve comparable healing rates; and functional and radiographic outcomes while reducing the related cost.

## Data Availability

The data that support the findings of this study are available from (Menoufia University Hospital) but restrictions apply to the availability of these data, which were used under license for the current study, and so are not publicly available. Data are however available from the authors upon reasonable request and with permission of (Menoufia University Hospital).

## Abbreviations

CRPS: Complex regional pain syndrome
DASH: Quick-Disabilities of the Arm, Shoulder and Hand
DRF: Distal radius fractures
IRUJ: Inferior radio-ulnar joint
IN-S: Intra-articular step-off
PT: Palmar tilt
RH: Radial height
RI: Radial inclination
ROM: Range of motion
SPSS: Statistical Package of Social Science
UV: Ulnar variance
